# Transdermal iontophoresis patch with reverse electrodialysis

**DOI:** 10.1080/10717544.2017.1282555

**Published:** 2017-04-20

**Authors:** Joon Lee, Kilsung Kwon, Minyoung Kim, Joonhong Min, Nathaniel S. Hwang, Won-serk Kim

**Affiliations:** 1 Biosensor Laboratories Incoperated, Seoul National University, Seoul, Republic of Korea,; 2 School of Chemical and Biological Engineering, Seoul National University, Seoul, Republic of Korea, and; 3 Department of Dermatology, Sungkyunkwan University School of Medicine, Kangbuk Samsung Hospital, Seoul, Republic of Korea

**Keywords:** Reverse electrodialysis, patch, iontophoresis, transdermal

## Abstract

Reverse electrodialysis (RED) technology generates energy from the salinity gradient by contacting waters with different salinity. Herein, we develop the disposable skin patch using this eco-friendly energy. The current density, which can be controlled easily without special circuit, is enough to iontophoretic drug delivery. *In vitro* study, this iontophoretic system enhanced the transdermal delivery of peptide, which is difficult to penetrate the skin barrier by simple diffusion. We design the disposable iontophoretic skin patch using RED system and suggest this patch can be apply on new cosmetic patch or disposable drug patch.

## Introduction

**Figure 1.**
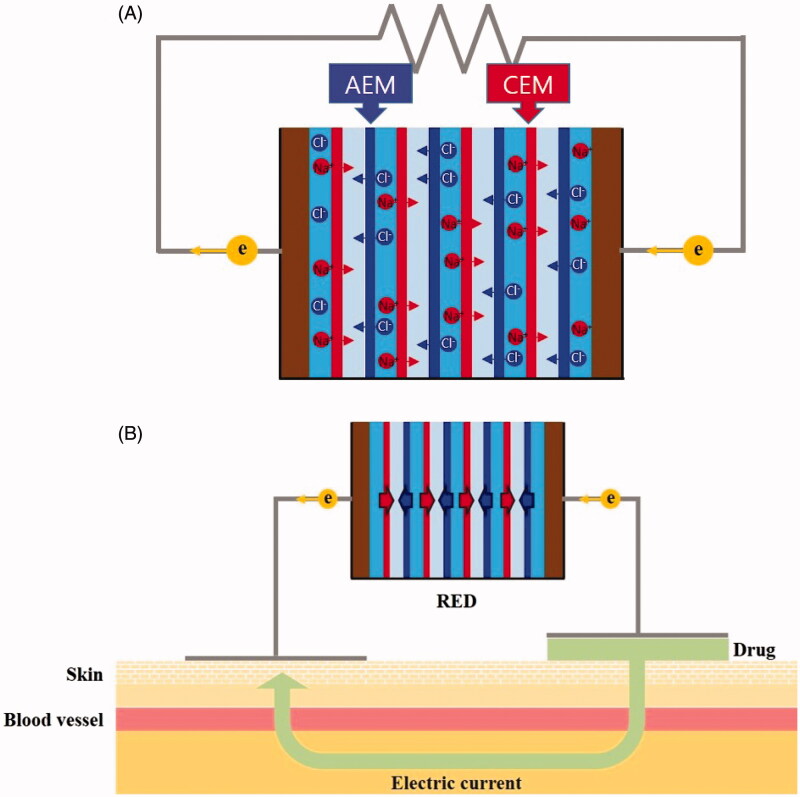
The principles of RED and iontophoretic drug delivery (A) the transport of cations and anions during the controlled mixing of saltwater and freshwater through selective ion exchange membranes (B) the net flow of drug solution from the anode to the cathode (or from the cathode to the anode according to charge of drug solution) under the influence of an electric current. AEM: anion exchange membrane; CEM: cation exchange membrane.

Reverse electrodialysis (RED) technology generates energy from the salinity gradient by contacting waters with different salinity, i.e. seawater and river water. This technique utilizes the transport of cations and anions during the controlled mixing of saltwater and freshwater through selective ion exchange membranes. The principle of RED has been well described in the literature (Pattle, 1954; Długołecki et al., 2008; Post et al., 2008; Veerman et al., 2009). A typical RED structure is a series of alternating cells determined by anion- and cation-exchange membranes in a stack placed between two electrodes (Figure 1(A)). Ion movement from the brine solution to the dilute solution is promoted by the salinity gradient. Since the membranes have selectivity for only specific types of ions (i.e. cation exchange membranes allow the passage of cations, and anion exchange membranes allow the passage of anions), cations are transported in the one direction and anions are transported in the other direction, resulting in a potential difference. Redox reactions then occur at the electrodes and the electro-neutrality of the solution in the electrode compartments can be maintained. Electrons flow from the anode to cathode via an external circuit, and this current can be used to power an external energy consumer.

Iontophoresis has been investigated and used as a tool for transdermal drug delivery for over 90 years, ever since Leduc showed that strychnine sulfate can be delivered to rabbits (Leduc, 1908). As peptide drugs were becoming available due to genetic engineering, iontophoretic drug delivery has gained new interest, since peptides are very difficult to deliver by conventional methods owing to their size and charge (Green et al., 1992; Green, 1996; Kanikkannan et al., 1999; Chang et al., 2000; Schuetz et al., 2005; Banga, 2007; Benson and Namjoshi, 2008; Herwadkar and Banga, 2011).

A skin patch using iontophoresis has been developed, but such electrically controlled patches require a connection to an external power source that is usually rigid, hazardous, non-disposable, and expensive (Kalia et al., 2004; Power, 2007; Dhaval et al., 2010; Calatayud-Pascuala et al., 2011; Vikelis et al., 2012; Saluja et al., 2013). Furthermore, a certain circuit is needed to control the electric energy at appropriate levels for iontophoresis without raising skin irritation or pain. In RED technology, electrical energy is easily controlled by adjusting the numbers and areas of the stacked cells. As the numbers and areas of cells increase, RED-producing current also tends to increase.

Herein, we designed the disposable iotophoresis patch, which use the electrical power generated from RED (Figure 1(B)). We evaluated the electrical performance of the RED batteries and studied the efficacy of the transdermal delivery by RED-driven iontophoresis.

## Materials and methods

### RED fabrication

**Figure 2.**
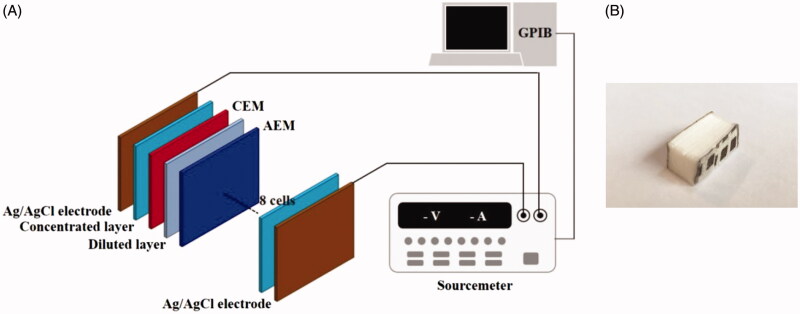
(A) Schematic of the RED system and experimental set up and (B) appearance of the disposable RED system.

Figure 2(A) shows the schematic diagram of a disposable RED and an experimental setup. The disposable RED was composed of electrodes, non-woven fabrics, cation exchange membranes (CEMs), and anion exchange membranes (AEMs). We used homogeneous Selemion^TM^ CMV and AMV membranes (Asahi Glass CO. Ltd., Japan). A non-woven fabric (SP60, NamYang Nonwoven Fabric CO. Ltd., South Korea) of 0.3 mm thickness was applied to both the concentrated layer and diluted layer. Sodium chloride (S5886, Sigma-Aldrich) was added to the concentrated layer to form a concentration gradient. The Ag/AgCl electrode was fabricated by electroplating. The base and both sides of the disposable RED were sealed with a typical epoxy to prevent leakage. The upper side was kept open to supply diluted solution. The active area for ion exchange was 2.4 cm × 0.6 cm, and the RED included 8 cell pairs (Figure 2(B)). A sourcemeter (2410, Keithley Instruments, INC.) operating in four-wire mode with source and sense wires (5806, Keithley Instruments, INC.) connected to the electrodes at the anode and cathode was employed to measure RED performance. It was connected to a computer using a GPIB-USB controller (GPIB-USB-HS, National Instruments) and was controlled with Labview 9.0 (National Instruments).

### Drugs

Fluorescein isothiocyanate labeled poly-l-lysine (FITC-PLL) were purchased from Peptron (Daejeon, South Korea), respectively.

### Preparation of skin

The porcine dorsal skins were obtained from commercially slaughtered pigs. On the day of experimentation, skin was thawed at room temperature and immediately cleaned with deionized water to remove hair and subcutaneous fat. Also, we purchased the pre-packed and commercialized micropig skin for diffusion cell (Medi Kinetics micropigs® Franz Cell Membrane, Pyungtaek, Kyunggido, South Korea) and stored at −80 °C until further use.

### Optical measurement of fluorescence distribution

Fluorophore distribution was optically measured from cross-sectioned skin samples. In order to compare the short-term transdermal fluorescence distribution of passive diffusion and the RED system, FITC-PLL solution (0.5 mM, 200 μL) was applied to porcine dorsal skins. The anode and cathode (conductive film, 4 cm^2^) of RED cells were attached across the pig skin. After a certain duration, the electrodes were detached and the skin was cleansed with wet tissue in order to eliminate residual signal. Thoroughly, washed skin specimens were fixed in 4% paraformaldehyde solution, immersed in 30% sucrose solution, and then placed into OCT compound and immediately frozen at −80 °C for sectioning. Vertical sections with 7 μm thickness were obtained using a Leica CM 1510S cryostat (Leica Biosystems, Germany), and the cryo-sectioned samples were mounted onto poly-l-lysine-coated slides. Covered with mounting solution, the specimens were observed using a Zeiss Axio Observer Z1 fluorescence microscope (Carl Zeiss MicroImaging GmbH, Göttingen, Germany).

### *In vitro* skin permeation studies

Excised miniature pig skin was examined using a modified Franz diffusion cell apparatus (Lab Fine Instruments, South Korea). The adjacent surface area was 12.19 cm^2^ and the receptor compartment volume was 26 ml and filled with isotonic phosphate buffered saline (pH 7.4) under 600 rpm of magnetic stirring. The temperature of each receptor part and the skin surface were kept at 37 ± 5 °C and 32 ± 5 °C, respectively. The skin was mounted onto the Franz cell directly and left an hour for membrane equilibration and hydration. Then, 300 μl of the 1 mM FITC-PLL solution was applied onto the surface of the skin. In order to compare efficacy, set-ups were categorized into two groups: passive diffusion and RED. In the case of RED experiments, an Ag/AgCl wire (anode) was dipped in the receptor compartment, while a conductive carbon film (4 cm^2^, cathode) was applied to the skin membrane. Both electrodes were connected to RED cells, which were activated by adding 2 ml of 11 mM sodium chloride electrolyte solution. In all experiments, the donor compartment of each Franz cell was covered with Parafilm® in order to prevent evaporation. At each predetermined time points (either 0.5, 1, 2, 3, 4, 5, 6, or 7 h), 500 μl aliquots were taken from the receptor compartment reservoir and stored at 4 °C prior to spectrophotometer analysis. The subtracted volume was replaced immediately by the same amount of fresh phosphate buffered solution. Each permeation experiment was conducted in triplicate or quadruplicate, and the concentrations of each sample were measured and calculated using a microplate reader (Tecan, Durham).

### Statistical data analysis

All data were expressed as mean ± S.E.M. and analyzed by two-tailed paired *t*-test or Wilcoxon matched-pairs signed-ranks tests. * = *p* < 0.05.

## Results

**Figure 3.**
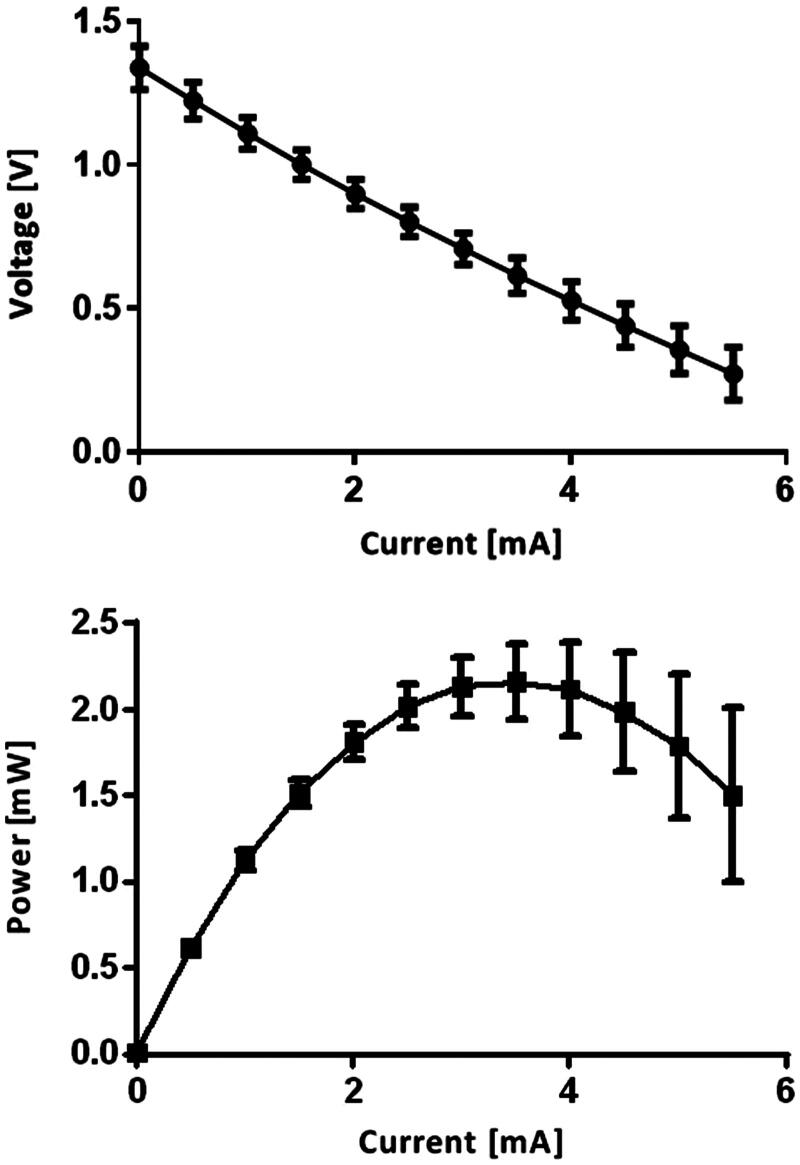
Polarization and power curves of the RED. *N_cell_ *= 8 and *C* = 0.01 M.

We first characterized the performance of the disposable RED at the peak point. Figure 2(A) shows the schematic diagram of a disposable RED in an experimental setup. Polarization and power density curves are shown in Figure 3. A 0.01 M NaCl solution was used as the diluted solution, and was supplied into the disposable RED from the top side using a pipette. It then permeated the non-woven fabric owing to capillary forces. A concentration gradient was formed by dissolving sodium chloride in the concentrated layer. We conducted our experiments at least four times for each data point to ensure the repeatability of the data. The error bars based on these independent realizations were estimated by the Student *t*-distribution with a 95% confidence interval using the following equation:

(1) $$x=\frac{\bar{x}+t\sigma }{\sqrt{N}}$$

where x, *t, σ*, and *N* are the mean, the *t*-distribution value, the standard deviation, and the number of experiments, respectively.

**Figure 4.**
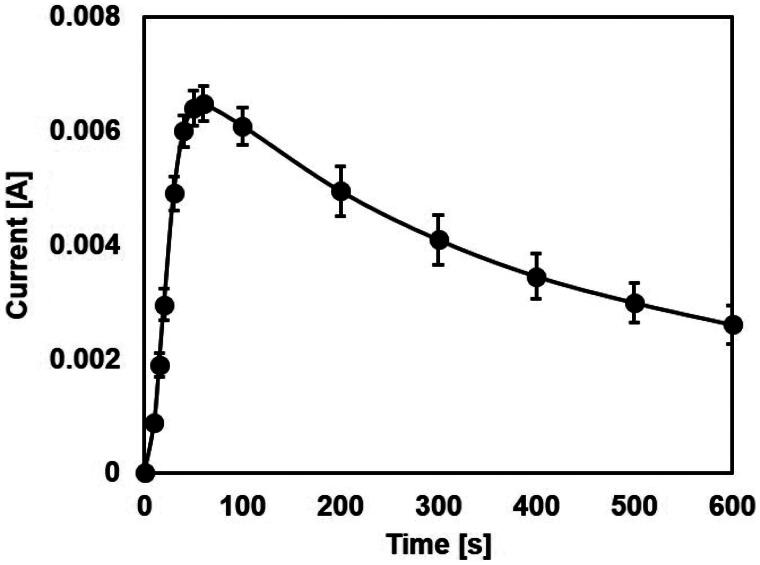
Time–current curves of the RED. *N_cell_ *= 8 and *C* = 0.01 M.

The voltage decreased as the current increased in a linear fashion, which was due to internal resistances, including the membrane resistance and the solution resistance in the RED. The polarization curve of the RED suggested the existence of ohmic loss only, which could be compared with the case of a fuel cell. The maximum voltage (zero current) was 1.33 V, and the maximum current (zero voltage) was 7.26 mA. We computed the power from the polarization curve using the simple formula *P *=* V*˙*I* where *P, V*, and *I* refer to the power, the terminal voltage, and current, respectively. The power curve had a parabolic shape with a maximum value of 2.5 mW. Maximum power occurred at half-maximum current produced from the RED. The RED-producing current reached maximum value with rapid increases within several minutes. After reaching maximum current, it slowly dropped and became undetectable within 1 hour (Figure 4).

**Figure 5.**
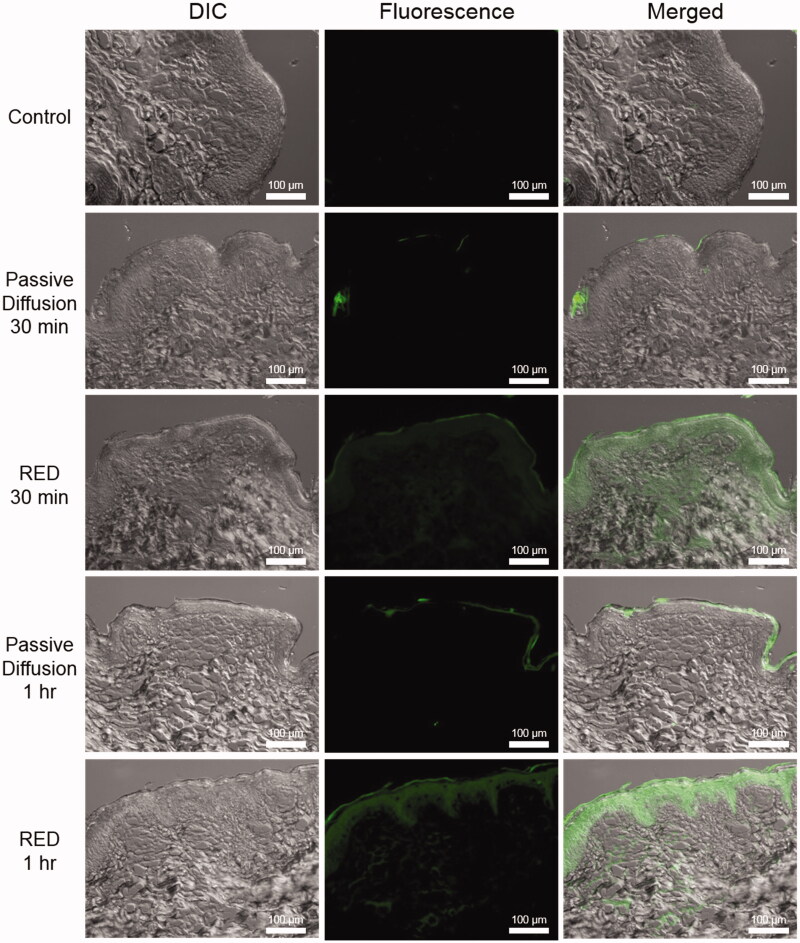
Distribution of FITC-PLL solution topically applied with or without RED on porcine skin. All images in this figure are magnified at 200×. Control, no treatment; passive diffusion, only topical application for 30 min and 1 h, respectively; RED, iontophoretic drug delivery for 30 min and 1 h, respectively (Left column, differential interference contrast (DIC) images; middle column, fluorescence images; right column, merged images).

To examine the feasibility of this RED-producing current, we preliminarily studied the RED-driven iontophoretic delivery of fluorescein isothiocyanate labeled poly-l-lysine (FITC-PLL). In this experiment, the distribution of fluorophores was optically measured from cross-sectioned skin samples. In order to compare the short-term transdermal fluorescence distribution of passive diffusion and the RED system, FITC-PLL solution (0.5 mM, 200 μL) was applied to porcine dorsal skin. The anode and cathode (conductive film, 4 cm^2^) of RED cells were attached across the pig skin. After durations of 30 min and 1 hour, as shown in Figure 5, the non-treated control experiment showed no fluorescent signal. In the passive diffusion model, the fluorescence was concentrated in the stratum corneum layer. However, in the RED experimental model, fluorescence was clearly observed in the dermis.

**Figure 6.**
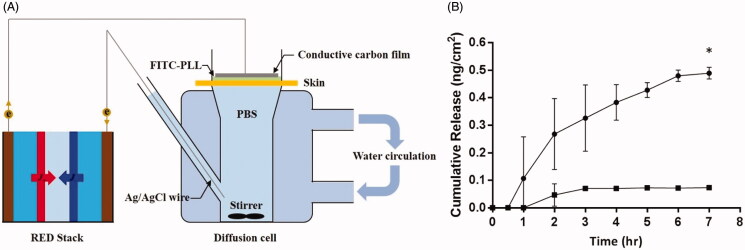
(A) Schematic of Franz diffusion cell apparatus (B) *In vitro* penetration of FITC-conjugated-PLL using a Franz diffusion cell equipped with micro pig skin with or without RED iontophoresis for 1 h. (▪; Passive diffusion, *n* = 3, •; RED iontophoresis, *n* = 2).

In this study, we compared the transdermal efficiency of passive diffusion and RED using a Franz diffusion cell apparatus (Figure 6(A)). FITC-labeled PLL was adopted as a peptide drug model due to its negative charge. It was applied for an hour and permeation was observed up to 7 hours. Quantification was based on the standard fluorescence curve of FITC-PLL. As shown in Figure 6(B), there was no great difference at first. However, the delivered FITC-PLL quantity in the passive diffusion and RED systems showed a drastic difference by 7 hours, with values of 0.0730 ± 0.002 ng/cm^2^ and 0.489 ± 0.021 ng/cm^2^, respectively. Therefore, it was concluded that after 7 h of operation, the RED system accelerated transdermal activity about 6.7 times more than the passive control system.

## Discussion and conclusions

The disposable iontophoresis patch using RED is composed of electrodes, non-woven fabrics, cation exchange membranes (CEMs), and anion exchange membranes (AEMs). We understand that the RED battery needs to be lightweight, as it needed to be applied to the skin. As such, we fixed the size of the RED battery at 24 × 9 × 6 millimeters with 8 pairs of cells. We used non-woven fabric with sodium chloride in order to create a sodium concentration gradient. An Ag/AgCl electrode was fabricated by electroplating. The base and both sides of the disposable RED were sealed with a typical epoxy to prevent leakage. The upper side was left open to supply diluted solution. After supplying the diluted solution to multi-stacked cells composed of AEM, non-woven fabric, and CEM, ion exchange was initiated.

We assumed that the current would decrease as the exchangeable ions in non-woven fabric with sodium chloride were exhausted. The current in the conventional RED is continuous and reproducible, as the brine and diluted water can be supplied continuously. In comparison to the conventional RED, we designed a small, disposable, and still eco-friendly RED battery lasting about 1 hour.

Iontophoresis implies the application of small amounts of physiologically acceptable current to drive drug molecules into and across skin. Two mechanisms, viz. electrorepulsion and electroosmosis, are primarily responsible for drug delivery enhancement by iontophoresis.

Electrorepulsion is predominantly responsible for the delivery of charged drug molecules across the skin. When a charged molecule is placed under an electrode of the same polarity, the repulsion between like charges drives the charged molecule through the skin. Electroosmosis is the net flow of water from the anode to the cathode under the influence of an electric current (Guy et al., 2000).

The use of iontophoresis for the systemic delivery of new peptides and protein therapeutic agents has attracted considerable recent interest from the biotechnology industry. Oral delivery results in protein denaturation in the acidic conditions of the stomach, as well as degradation by gastrointestinal enzymes, leading to poor bioavailability. Therefore, the ability of iontophoresis to deliver drugs of relatively high molecular weights in a controlled and reliable fashion is an issue of great importance.

After activating the RED by applying diluted solution on the upper side of RED, the ionic current with electro-osmosis moved from the anode to the cathode. Active ingredients, such as drugs and cosmetics, can thus be administrated into the skin.

We tested whether a RED-producing current could maintain an open circuit on porcine skin. We measured the current density through porcine skin. The maximum current density was 260 μA/cm^−2^ through porcine skin, and the resistance was ∼5 kΩ (data not shown). As a reference point, the resistance of human skin on the arms and the backs of hands for three men was 270–360 Ω. We assumed that the RED-producing current can be used for the iontophoresis of drugs or chemicals.

FITC-PLL has a negative charge with variable molecular weight; we synthesized it at ∼1000 g/mol. It is well-known as chemicals which have difficultly penetrating the skin barrier via passive diffusion due to their size and charge (Turner et al., 1997).

It is therefore concluded that the RED system allows enhanced transdermal drug delivery within a short time relative to passive diffusion, which implies a simple application of drugs on the skin. Thus, we suggest that the herein developed RED system may be used practically for transdermal drug delivery or as a functional cosmetic patch.

In conclusion, the current generated from RED has been successfully used in iontophoretic drug delivery. Although molecular weight of chemicals, we used was relatively high (> 500 g/mol), transdermal delivery was still enhanced significantly. The RED system we modified was cheap, eco-friendly, and disposable, because it is composed of only fabric, salt, and ion exchange membranes. We propose that RED-producing current should be available for an iontophoretic cosmetic or drug patch.
